# Long-Term *in vivo* Evaluation of Orthotypical and Heterotypical Bioengineered Human Corneas

**DOI:** 10.3389/fbioe.2020.00681

**Published:** 2020-06-19

**Authors:** Ingrid Garzón, Jesus Chato-Astrain, Carmen González-Gallardo, Ana Ionescu, Juan de la Cruz Cardona, Miguel Mateu, Carmen Carda, María del Mar Pérez, Miguel Ángel Martín-Piedra, Miguel Alaminos

**Affiliations:** ^1^Tissue Engineering Group, Department of Histology, University of Granada, Granada, Spain; ^2^Instituto de Investigación Biosanitaria ibs.GRANADA, Granada, Spain; ^3^Division of Ophthalmology, University Hospital San Cecilio, Granada, Spain; ^4^Biomaterials Optics Group, Department of Optics, University of Granada, Granada, Spain; ^5^Department of Histology and Pathology, University of Valencia, Valencia, Spain

**Keywords:** tissue engineering, bioengineered cornea, Wharton’s jelly stem cells, heterotypical human cornea, artificial cornea

## Abstract

**Purpose:**

Human cornea substitutes generated by tissue engineering currently require limbal stem cells for the generation of orthotypical epithelial cell cultures. We recently reported that bioengineered corneas can be fabricated *in vitro* from a heterotypical source obtained from Wharton’s jelly in the human umbilical cord (HWJSC).

**Methods:**

Here, we generated a partial thickness cornea model based on plastic compression nanostructured fibrin-agarose biomaterials with cornea epithelial cells on top, as an orthotypical model (HOC), or with HWJSC, as a heterotypical model (HHC), and determined their potential *in vivo* usefulness by implantation in an animal model.

**Results:**

No major side effects were seen 3 and 12 months after implantation of either bioengineered partial cornea model in rabbit corneas. Clinical results determined by slit lamp and optical coherence tomography were positive after 12 months. Histological and immunohistochemical findings demonstrated that *in vitro* HOC and HHC had moderate levels of stromal and epithelial cell marker expression, whereas *in vivo* grafted corneas were more similar to control corneas.

**Conclusion:**

These results suggest that both models are potentially useful to treat diseases requiring anterior cornea replacement, and that HHC may be an efficient alternative to the use of HOC which circumvents the need to generate cornea epithelial cell cultures.

## Introduction

Transplantation of a functional tissue-engineered cornea could contribute to the clinical treatment of patients with severe corneal defects (Garzon et al., 2014b; Gonzalez-Andrades et al., 2017). Several approaches have been described for the treatment of diseased corneas from a regenerative medicine standpoint (Oie and Nishida, 2016). On the one hand, tissue-engineered cell sheets or individual corneal layers can be efficiently generated in the laboratory using corneal epithelial cells (Sangwan and Sharp, 2017; Pellegrini et al., 2018; Le-Bel et al., 2019), stromal keratocytes (Isaacson et al., 2018) or extra-corneal cells (Nishida et al., 2004) combined with different types of biomaterials or substrates. On the other hand, promising models of bioartificial corneas have been developed by tissue engineering (Ghezzi et al., 2015), including full-thickness corneas (Griffith et al., 1999; Alaminos et al., 2006) and anterior lamellar partial thickness corneas consisting of an epithelial layer with cornea epithelial cells and a subjacent stromal layer with biomaterials and keratocytes (Gonzalez-Andrades et al., 2009; Mi et al., 2010; Zaniolo et al., 2013; Couture et al., 2016; Rico-Sanchez et al., 2019).

However, the clinical translation potential of most of these strategies is still uncertain and highly dependent on the availability of adequate limbal stem cell sources for the generation of bioengineered cornea models with corneal cells. In this regard, human limbal stem cell cultures raised from limbal biopsies are difficult to obtain, and the procedure is subjected to the risk of causing limbal stem-cell deficiency in the healthy eye (Nishida et al., 2004).

In addition, patients suffering from bilateral limbal stem cell deficiency (LSD) may not be candidates for limbal biopsy and need alternative sources of corneal epithelial cells (Nguyen et al., 2017). For these reasons, the search for alternative cell sources to generate heterotypical bioartificial human corneas using extra-corneal cells is a current challenge in tissue engineering.

Previous reports have demonstrated that human mesenchymal stem cells (MSC) may have the potential to differentiate into cornea epithelial cells for the generation of partial human corneas *in vitro* (Liu et al., 2008; Gomes et al., 2010; Lin et al., 2013). Among the different types of MSC that could be potentially used in cornea tissue engineering, human umbilical cord Wharton’s jelly stem cells (HWJSC) have several advantages, including accessibility, proliferation and differentiation potential, and immune-privileged status (Garzon et al., 2020). In fact, our research group was able to generate *in vitro* a biomimetic substitute of the human anterior cornea using HWJSC as an alternative cell source (Garzon et al., 2014b). Although promising results were obtained *in vitro*, the *in vivo* usefulness of these bioengineered corneas remains to be determined.

In the present study, we generated partial human orthotypical cornea (HOC) models with corneal cells, and human heterotypical cornea (HHC) models with HWJSC. We then characterized the main stromal and epithelial markers of these bioengineered cornea substitutes both *in vitro* and *in vivo* to determine the differentiation potential of HWJSC and the clinical translational potential of bioengineered corneal substitutes with this source of stem cells.

## Materials and Methods

### Cell Isolation and Culture

To generate primary cultures of human cornea stromal and epithelial cells, we used limbal sclero-cornea rings that were discarded after cornea transplant surgery. First, cornea remnants attached to the limbus were isolated by surgical dissection and digested in *Clostridium histolyticum* type-I collagenase (Thermo Fisher Scientific, Waltham, MA, United States) for 6 h at 37°C. Isolated keratocytes were obtained by centrifugation and cultured in Dulbecco’s modified Eagle’s medium (DMEM) supplemented with 10% fetal calf serum, 4 mM L-glutamine and 1% antibiotic–antimycotic solution (Thermo Fisher Scientific). Then the limbal tissue was carefully dissected and small explants were cultured directly in Petri dishes with epithelial cell culture medium (Garzon et al., 2014b). HWJSC were isolated from five human umbilical cords obtained from cesarean deliveries using published protocols (Garzon et al., 2014a). First, arteries and veins were removed from the umbilical cord, and the Wharton’s Jelly tissue was surgically dissected and digested with a mixture of type-I collagenase (Thermo Fisher Scientific) and a 0.5 g/L trypsin – 0.2 g/L EDTA solution (Sigma-Aldrich, St. Louis, MO, United States). Cells were harvested by centrifugation and cultured in 75 cm^2^ culture flasks using Amniomax^TM^ culture medium (Thermo Fisher Scientific). All cell cultures were kept in a cell incubator at 37°C with 5% CO_2_ using standard cell culture conditions.

All methods and experimental protocols were performed in accordance with relevant guidelines and regulations according to the Association for Research in Vision and Ophthalmology (ARVO) Statement for the Use of Animals in Ophthalmic and Vision Research. For the use of human tissues, the project was approved by the local Human Research and Ethics Committee of the province of Granada -PEIBA- (numbers 9/2017 and 3/2016), and all tissue donors or their parents and/or legal guardians provided their informed consent.

### Generation of HOC and HHC Cornea Models

Orthotypical models of the human cornea containing cornea cells (HOC) and heterotypical models containing non-corneal cells as the epithelial cell source (HHC) were generated with fibrin-agarose biomaterials with a final agarose concentration of 0.1% as previously reported by our research group (Gonzalez-Andrades et al., 2009; Garzon et al., 2014b). To do so, we first fabricated a biomaterial containing corneal stromal cells that will act as a biological substitute of the corneal stroma, and we then generated an epithelial layer (with corneal or extra-corneal cells) on top of this stroma substitute (Alaminos et al., 2006; Gonzalez-Andrades et al., 2009; Ionescu et al., 2015) to resemble the structure of the human native cornea. To generate the stromal substitute, we used the following protocol: per each ml of mixture, 760 μl of human plasma (obtained from healthy blood donors) were mixed with 15 μl of tranexamic acid (Amchafibrin, Fides-Ecofarma, Valencia, Spain), 75 μl of DMEM (Thermo Fisher Scientific) containing 100,000 cultured stromal cells (activated fibroblasts), 50 μl of melted 2% type-VII agarose in PBS (both, from Sigma-Aldrich) and 100 μl of 1% CaCl_2_ (Sigma-Aldrich). This mixture was rapidly aliquoted in Transwell cell culture inserts with 0.4 μm porous membranes (Corning-Costar, Corning, NY, United States) and allowed to jellify at 37°C for at least 6 h. Then, HOC models were generated by subculturing the previously cultured cornea epithelial cells on top of the stroma substitutes, whilst HHC models were generated with HWJSC instead of epithelial cells. In both models, stratification and differentiation of the epithelial-like cell layer were promoted with an air–liquid culture technique. This technique was performed by lowering the amount of culture medium in the porous inserts, so that only the stromal substitute remained submerged, whereas the developing epithelium was in direct contact with air and received nutrition from the stromal layer (Reichl and Muller-Goymann, 2003).

Finally, bioengineered corneas were subjected to plastic compression nanostructuration (Ionescu et al., 2011). This technique was previously used to induce the formation of irreversible covalent interactions and hydrogen bonding between the molecules of the biomaterial, resulting in a significant improvement of the biomechanical properties of these artificial tissues that allowed surgical handling (Ionescu et al., 2011; Campos et al., 2016). For this, bioengineered corneas were placed between a pair of sterile nylon filter membranes (Merck-Millipore, Darmstadt, Germany), and two pieces of Whatman 3 mm absorbent paper (Sigma-Aldrich) were set in contact with each filter membrane. Then, a flat glass surface weighing 500 g was used to apply a homogeneous pressure on the surface of the system for 3 min. Finally, human bioartificial corneas were carefully retired and kept in PBS to prevent them from excessive dehydration.

### *In vivo* Evaluation of HOC and HHC Cornea Models in Laboratory Animals

Animal study design: in order to characterize both cornea models *in vivo*, samples were implanted on the anterior cornea surface of New Zealand laboratory rabbits using the DALK technique. A total of 20 rabbits were included in the study, which were randomly assigned to one of the following groups: (1) four animals were grafted with HOC and euthanatized after 3 months of follow-up, (2) four animals with HOC analyzed after 12 months, (3) four animals with HHC analyzed after 3 months, (4) four animals with HHC analyzed after 12 months, and (5) four non-operated rabbits used as controls.

Animals were deeply anesthetized by an intramuscular injection of xylazine (5 mg/kg) followed 10 min later by an intramuscular injection of ketamine (25 mg/kg), and local anesthetics drops were administered in the eye. Then the right eye was exposed, and the anterior layers of the cornea (approximately, two thirds of the stromal thickness) were surgically removed using a surgical microscope. To do that, a manual keratotome was first used to make a circular incision approximately 6 mm in diameter and 200 μm deep in the corneal thickness. Then an air bubble was injected into the central layer of the cornea stroma with a Hessburg-Barron vacuum trephine, and an ophthalmic crescent knife was used to horizontally dissect the most external stromal layers along with the superjacent epithelium. The limbus was preserved in all eyes. After careful debridement of the cornea surface, the bioengineered HOC or HHC was placed on this surface and sutured to the remaining cornea with 10/0 nylon suture stitches. Local antibiotics and anesthetics drops were administered after surgery. To protect the artificial tissue from mechanical damage, the eyelid of the treated eye was closed for 1 week. In all animals, the left eye was not treated and served as a control.

Ophthalmic evaluation was carried out every week, and slit-lamp bio-microscopy and anterior segment optical coherence tomography (OCT) was performed at the moment of the euthanasia of each animal (3 and 12 months of the surgical procedure). Both eyes were examined in each animal, and major side effects were registered, including tumorigenesis, hemorrhage, rejection or infection. The presence of inflammation was identified by the presence of hyperemia or neovascularization. Then, all eyes were surgically extracted and analyzed histologically as described below.

Animal experimentation was approved by the regional ethical committee (Ethics and Animal Experimentation Committee of the Andalusian Directorate of Agricultural Production – CEEA), ref. number 25/06/2018/099.

### Histological and Immunohistochemical Evaluation of HOC and HHC Cornea Models

Histological methods were used to evaluate *in vitro* HOC and HHC samples corresponding to 1, 2, and 3 weeks of development and *in vivo* HOC and HHC samples grafted in laboratory animals for 3 and 12 months. Samples were fixed in 3.7–4.0% wt/vol buffered formaldehyde for 24 h at 4°C and dehydrated in ethanol series (1 h in 70% ethanol, two incubations of 1 h in 96% ethanol, two incubations of 1 h in 100% ethanol), cleared in xylene (two incubations of 1 h) and embedded in paraffin (two incubations of 1 h in paraffin melted at 60°C) using an automated Citadel Tissue Processor (Thermo Fisher Scientific). Tissue sections 5 μm thick were obtained and placed on glass slides, dried at room temperature, dewaxed with three incubations of 5 min in xylene and rehydrated with an ethanol series (three incubations of 5 min in 100%, two incubations of 5 min in 96%, 5 min in 70%, 5 min in 50% ethanol, and 5 min in distilled water).

For histological analysis, tissue sections were incubated for 3 min in hematoxylin (PanReac AppliChem, Barcelona, Spain), rinsed in tap water for 5 min, stained with eosin (PanReac AppliChem) for 1 min and dehydrated using an ethanol series (3 incubations of 5 min in 96% and 3 incubations of 5 min in 100% ethanol), xylene (3 incubations of 15 min) and cover-slipped using Permount (Fisher Scientific International, Pittsburgh, PA, United States). Tissue sections stained with H&E were used to quantify the number of cell layers in the corneal epithelium. In each sample, the epithelial layer was examined using 600× magnification, and the number of cell layers was determined by three expert histologists.

Immunofluorescence assays in tissue sections were performed using previous standardized protocols (Martin-Piedra et al., 2017). Samples were first treated for 25 min with 0.01 M citrate buffer (Dako, Glostrup, Denmark) at 95°C for antigen retrieval. After rising in PBS, samples were preincubated in prehybridization buffer containing 10% donkey serum, and primary antibodies were applied and incubated for 2 h at room temperature. Then, samples were washed three times in PBS and secondary FITC-conjugated anti-mouse or Cy3-anti-rabbit secondary antibodies (Sigma-Aldrich) were used at 1:500 dilution. Tissues were then counterstained using DAPI mounting medium (Vector Laboratories, Burlingame, CA, United States) and cover-slipped. Analysis was performed using a Nikon Eclipse 90i fluorescence microscope. Primary antibodies used for immunofluorescence analysis were specific of the cornea epithelium: anti-cytokeratin 3/12 (Abcam ab68260), plakoglobin (Abcam ab12083), crystallins CRY-αA (Santa Cruz sc-28306), CRY-αB (Millipore ABN185), CRY-β (Santa Cruz sc-22745), CRY-λ1 (Sigma Prestige HPA040403), and CRY-Z (Abcam ab154842). As the manufacturers claims that anti-PKG and anti-CRY-λ1 antibodies are specific for human antigens, these were therefore used to specifically identify human cells. The immunofluorescence results were analyzed semiquantitatively by three independent histologists in a blinded manner to reduce potential biases. Each histologist scored the positive signal in each sample as strong (+++), moderate (++), slight (+), very slight (±) or negative (−), as previously described (Garzon et al., 2009).

For immunohistochemistry, tissue sections were treated with 3% H_2_O_2_ to quench endogenous peroxidase, and antigen retrieval and prehybridization were carried out as described for immunofluorescence. Then, samples were incubated overnight at 4°C in anti-type I collagen (Acris R1038) or anti-vimentin (Sigma V6630) primary antibodies and washed in PBS. Then, peroxidase-labeled secondary antibodies (Vector Laboratories) were used and the signal was detected using a diaminobenzidine (DAB) development kit (Vector Laboratories). In both cases (immunofluorescence and immunohistochemistry), isotype-specific immunoglobulins were used instead of the primary antibodies as negative controls.

For scanning electron microscopy (SEM), samples were fixed in cacodylate-buffered 2.5% glutaraldehyde, dehydrated in alcohol series (50, 70, 96, and 100% ethanol, 30 min in each concentration) and dried by the critical point method using a Critical Point Dryer (Bal-Tec, Los Angeles, CA, United States). This method consists in replacing all the ethanol embedding the tissue by liquid carbon dioxide at high pressure, which is then evaporated at low pressure, resulting in a critically dried sample. Then, tissues were sputter-coated with gold-palladium using a Mini Sputter Coater System (Quorum Technologies Ltd., Lewes, United Kingdom) and examined a Quanta 200 scanning electron microscope (FEI, Eindhoven, Netherlands).

For transmission electron microscopy (TEM), samples were fixed in cacodylate-buffered 2.5% glutaraldehyde for 8 h, washed three times in cacodylate duffer and postfixed in 1% osmium tetroxide for 90 min. After fixation, samples were dehydrated in increasing concentrations of acetone (30, 50, 70, 95, and 100%), embedded in Spurr’s resin and cut into ultrathin sections with an ultramicrotome. For analysis, sections were stained with aqueous uranyl acetate and lead citrate and examined with an EM902 transmission electron microscope (Carl Zeiss Meditec, Inc., Oberkochen, Germany).

### Optical Properties of the HOC and HHC Cornea Models

In order to characterize the different cornea models from an optical standpoint, we analyzed the transmittance of each sample and the reduced scattering and absorption coefficients. The inverse adding-doubling method was used to determine scattering and absorption using total reflection and total transmission measurements made with a single integrating sphere, as previously described (Ionescu et al., 2015, 2017). Total diffuse reflection and transmission measurements were made with a 158.2-mm-diameter integrating sphere (Oriel, model 70674. Newport Corp., Irvine, CA, United States) with a 11-mm-diameter detector port and a 4-mm-diameter sample port with a baffle between ports. The entrance port was 15 mm in diameter. All measurements were obtained for HOC and HHC corneas and native control corneas at 457.9, 488, and 514.5 nm with an argon ion laser (Stellar-Pro-L Model, Modu-Laser, Centerville, UT, United States) and at 632.8 nm with a He-Ne laser (30564 Model, Research Electro-Optics, Boulder, CO, United States). The maximum output power was 1000 mW ± 5% for the argon laser and 12 mW for the He-Ne laser. The diameter of both argon and He-Ne lasers beams was 2 mm. Three reflection measurements were made in each sample, and were referenced to a 98% Optopolymer reflectance standard (OPST3-C, Optopolymer, Munich, Germany) and a dark measurement (with the sample port empty). For transmission, three measurements were made in each corneal model and a control cornea, and were referenced to 100% with the lasers illuminating the open port (empty port) and a dark measurement with an open port but with no illumination from the lasers. Reflectance of the sphere wall, thickness, and refractive index of the cornea models and the control native cornea were also measured and used to calculate the absorption and reduced scattering coefficients, using the inverse adding-doubling method (Prahl et al., 1993; Sardar et al., 2005, 2009; Yust et al., 2012).

### Statistical Analysis

Quantitative results obtained for the optical parameters (transmittance, absorption and reduced scattering) were analyzed statistically to identify differences among samples. First, a global comparison of all study samples (HOC, HHC, and controls) was carried out using the Kruskal–Wallis test. Then, *post hoc* comparisons between two specific types of corneas (HOC vs. controls, HHC vs. Controls, and HOC vs. HHC) was performed using the Mann–Whitney test. Non-parametric tests were used because the parameters analyzed here did not fulfill the criteria for parametric testing. A Bonferroni-adjusted *P*-value of 0.001 was considered as statistically significant for the double-tailed tests, since numerous statistical comparisons were carried out at the same time. *P*-values between 0.05 and 0.001 were considered as marginally significant, meaning that these values would have been considered as significant if the Bonferroni correction had not been applied.

## Results

### Clinical Results

**FIGURE 1:**
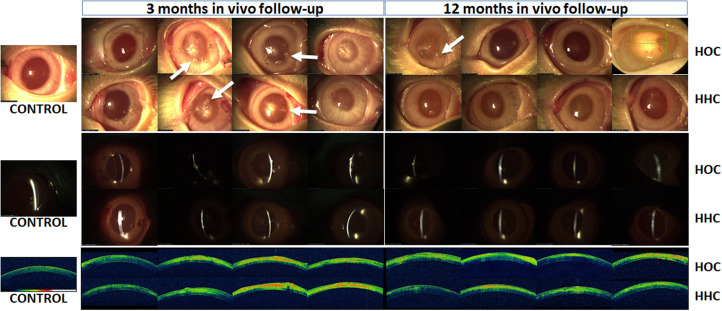
Clinical results in HOC and HHC bioartificial corneas grafted on the surface of rabbit eyes after 3 and 12 months of follow-up. Top images show the macroscopic findings; central images show slit lamp observations; lower images illustrate OCT findings. Controls are non-grafted normal rabbit eyes. HOC, human orthotypical cornea; HHC, human heterotypical cornea; OCT, optical coherence tomography. Arrows correspond to the inflammatory signs around the suture stitches.

*In vivo* analyses of generated HOC and HHC models allowed us to evaluate the safety and biointegration of bioartificial corneas. In this regard, we found no signs of tumorigenesis, hemorrhage, rejection, infection, or other major side effects in any of the animals. Macroscopic examination of the rabbit eyes revealed that HOC and HHC resulted in corneas with some inflammatory signs around the suture stitches, especially 3 months after *in vivo* implantation. However, inflammation tended to resolve after 12 months. In fact, ophthalmic evaluation of HOC revealed some inflammation around the stitches in 2 out of the 4 rabbits analyzed at 3 months, and 1 out of 4 rabbits at 12 months. For HHC, 2 of the 4 animals showed inflammation at 3 months, and none at 12 months (Figure 1). Slit lamp analyses showed no differences between the two types of cornea and the controls, with a well-defined linear structure of the cornea in all cases. OCT evaluation revealed a regular structure consisting of a corneal stroma with a normal overlying epithelium in control eyes. Cross-sectional images obtained by OCT on corneas undergoing surgery showed that the grafted tissue was tightly attached to the host cornea stroma in all cases. However, an interface between both tissues was clearly identifiable in all HOC and HHC eyes after 3 and 12 months (Figure 1).

### Histological Analysis

**FIGURE 2:**
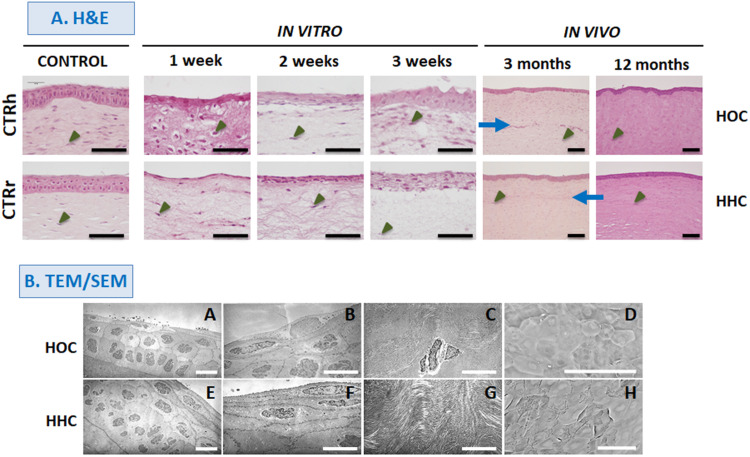
Histological evaluation of different samples with hematoxylin and eosin (H&E) staining **(A)**, and electron microscopy **(B)**. HOC, human orthotypical cornea; HHC, human heterotypical cornea. A, B, E, and F show TEM images of HOC and HHC epithelium at different magnifications. C and G show TEM images of corneal stroma. D and H show SEM images of the corneal epithelial surface in HOC and HHC. Controls are normal human corneas (CTRh) and normal rabbit corneas (CTRr). Blue arrows correspond to the interface between the host tissue and the implant, and some illustrative stromal cells are highlighted with arrow heads. Scale bars: 100 μm for H&E and SEM, and 10 μm for TEM images.

Histological evaluation of different samples by H&E are shown in Figure 2A. Analyses of *in vitro* HOC samples showed a stratified epithelium consisting of polygonal and rounded cells. Quantification of the number of cell layers disclosed that the cell number tended to increase with time in culture: at 1 week, HOC had an average of 2 epithelial cell layers, at 2 weeks, we found an average of 4.5 layers, and an average of 6 layers were found at week 3. Analysis of *in vitro* HHC samples showed that these cells tended to be more flattened as compared to HOC, and showed an average of 1.5 cell layers at week 1, 6.5 layers at week 2, and 9 layers at week 3. Regarding the stromal cells, we found that these cells were scattered throughout the fibrin-agarose biomaterial in all sample types.

*In vivo* grafting resulted in higher levels of tissue differentiation in both types of cornea. In all *in vivo* samples, the epithelium consisted of an average of 6.5 epithelial cell layers showing a well-organized stratification pattern very similar to control corneas. Moreover, the stroma was formed by a dense, highly organized fibrillar mesh containing large numbers of stromal cells after 12 months of development, whereas the interface between the host tissue and the implant was detectable only in samples corresponding to 3 months after *in vivo* implantation.

Further characterization of the *in vivo* HOC and HHC samples with TEM (Figure 2B) confirmed the presence of a stratified corneal epithelium that showed polarity and signs of differentiation such as rounded cells in the basal layer and flattened cells on top, cell–cell cohesion, and a basement membrane underlying the corneal epithelium. In the stroma, abundant collagen fibers were found with different spatial orientation corresponding to different corneal stroma layers, and some stromal cells were detected between adjacent stromal layers.

Analyses with SEM (Figure 2B) showed that the surface of HOC and HHC was fully covered by an epithelial layer consisting of flat polygonal cells exhibiting evident signs of cell desquamation and cell renewal.

### Corneal Stroma Differentiation Determined by Immunohistochemistry

**FIGURE 3:**
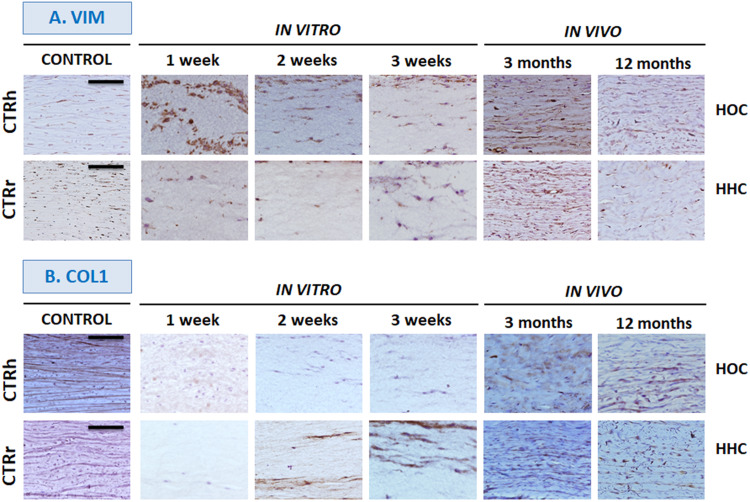
*In vitro* and *in vivo* analyses of corneal stroma differentiation determined by immunohistochemistry for vimentin (VIM, **A**) and type I collagen (COL1, **B**). HOC, human orthotypical cornea; HHC, human heterotypical cornea. Controls are normal human corneas (CTRh) and normal rabbit corneas (CTRr). Scale bars: 100 μm for all images.

Analyses of cells in the stroma of different samples showed that most cells were positive for the vimentin stromal marker both *in vitro* and *in vivo* (Figure 3A). In addition, collagen I immunohistochemistry (Figure 3B) showed that HOC maintained *in vitro* did not express this marker, but collagen was present in *in vivo* samples at 3 months and its expression was increased at 12 months. In contrast, HHC showed positive collagen expression after 2 weeks of *in vitro* development, and expression increased steadily thereafter. The highest levels of expression were seen *in vivo*, especially after 12 months being similar to control group (Figure 3B).

### Corneal Epithelium Differentiation Determined by Immunofluorescence

**FIGURE 4:**
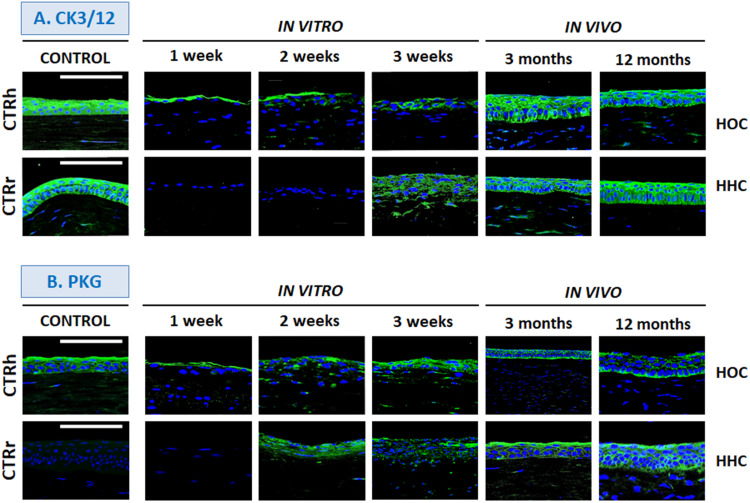
Differentiation of corneal epithelium *in vitro* and *in vivo* determined by immunohistochemistry for cytokeratin 3/12 (CK3/12, **A**) and plakoglobin (PKG, **B**). HOC, human orthotypical cornea; HHC, human heterotypical cornea. Controls are normal human corneas (CTRh) and normal rabbit corneas (CTRr). Scale bars: 100 μm.

To determine the epithelial phenotype of the epithelial layer in HOC and HHC cornea substitutes, we analyzed CK3/12 and PKG expression. Orthotypical epithelial cells produced positive signals for CK3/12 cytokeratin in all stages of *in vitro* development, especially in the most superficial layer (Figure 4A and Table 1). This expression increased and showed strong positivity *in vivo* and was seen in all epithelial layers, being comparable to controls. However, HHC were initially negative and turned moderately positive after 3 weeks of *in vitro* development. In our *in vivo* samples of HOC and HHC, expression levels were strong and similar to those in control human corneas.

**TABLE 1:** Semiquantitative analysis of expression of several epithelial markers as determined by immunofluorescence.

		*In vitro* 1 week	*In vitro* 2 weeks	*In vitro* 3 weeks	*In vivo* 3 months	*In vivo* 12 months	Control human cornea	Control rabbit cornea
CK3/12	HOC	+	+	++	+++	+++	+++	+++
	HHC	−	−	++	+++	+++		
PKG	HOC	+	+	+++	+++	+++	+++	±
	HHC	−	++	++	+++	+++		
CRY-αA	HOC	+	++	+++	+++	+++	+++	+++
	HHC	+	+	+++	+++	+++		
CRY-αB	HOC	−	±	+	±	++	+++	+++
	HHC	±	+	+	+	+++		
CRY-β	HOC	+	+	++	++	++	++	++
	HHC	−	+	+	++	+		
CRY-λ1	HOC	++	++	++	+	+	+++	±
	HHC	++	++	++	+	++		
CRY-Z	HOC	−	+	++	++	++	+++	+
	HHC	+	++	+++	++	+++		

*HOC, human orthotypical cornea; HHC, human heterotypical cornea.*

Analysis of the cell–cell junction protein PKG (Figure 4B and Table 1), also showed positive expression in all HOC samples, especially at week 3 *in vitro* and in all *in vivo* samples, which showed strong expression. For the HHC samples, *in vitro* corneas were moderately positive for PKG from week 2 of development, and *in vivo* samples were strongly positive. Interestingly, PKG expression was very low in control rabbit corneas and strongly positive in control human tissues.

### Crystallin Expression

**FIGURE 5:**
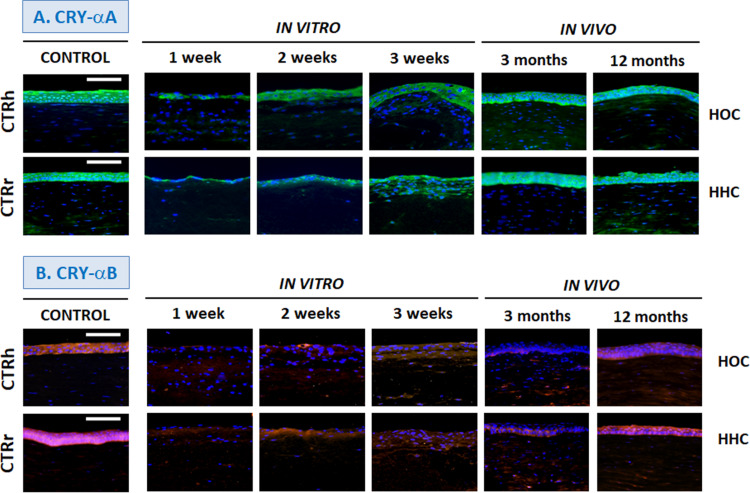
Differentiation of corneal epithelium *in vitro* and *in vivo* determined by immunohistochemistry for crystallins CRY-αA **(A)** and CRY-αB **(B)**. HOC, human orthotypical cornea; HHC, human heterotypical cornea. Controls are normal human corneas (CTRh) and normal rabbit corneas (CTRr). Scale bars: 100 μm.

**FIGURE 6:**
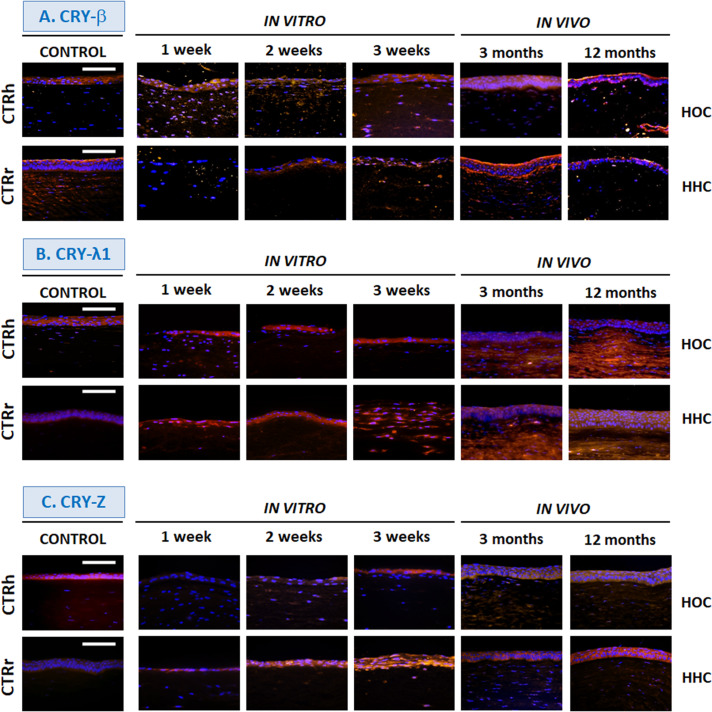
Differentiation of corneal epithelium *in vitro* and *in vivo* determined by immunohistochemistry for crystallins CRY-β **(A)**, CRY-λ1 **(B)** and CRY-Z **(C)**. HOC, human orthotypical cornea; HHC, human heterotypical cornea. Controls are normal human corneas (CTRh) and normal rabbit corneas (CTRr). Scale bars: 100 μm.

Immunohistochemical assays for crystallins, key proteins in corneal transparency, showed that all proteins were highly expressed by the control human cornea epithelium, although control rabbit cornea showed slight or very slight expression levels of CRY-Z and CRY-λ1 (Figures 5, 6 and Table 1). In the bioengineered corneas, expression of all crystallins tended to increase with time in all corneas maintained *in vitro*, with highest levels in corneas with the highest number of epithelial cell layers except for CRY-λ1, which was moderately positive from week 1 (Figure 6B and Table 1). In general, the expression levels tended to be higher in *in vitro* HOC samples than in *in vitro* HHC samples, except for CRY-Z (Figure 6C and Table 1), which was expressed more strongly in HHC than HOC, and CRY-λ1, which was expressed at similar levels in both models. After 3 months of *in vivo* development, HOC and HHC samples were comparable to control human corneas for CRY-αA (Figure 5A and Table 1) and CRY-β (Figure 6A and Table 1), whereas lower expression was found for the rest of crystallins analyzed in this work. At 12 months of *in vivo* development, the levels of CRY-αA expression in both HOC and HHC were strong and similar to human control cornea, and this was also the case of CRY-αB (Figure 5B and Table 1) and CRY-Z for the HHC samples. Controls *in vivo* HOC samples at 12 months showed similar moderate expression. However, *in vivo* expression levels of CRY-αB, CRY-λ1, and CRY-Z were lower in HOC samples at 12 months as compared to human controls, and HHC samples at 12 months showed lower CRY-β and CRY-λ1 expression levels than human controls.

### Optical Behavior

**FIGURE 7:**
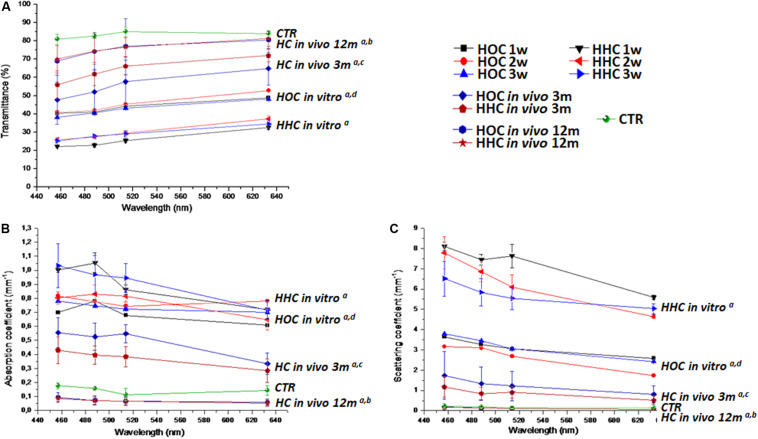
Optical properties of cornea models *in vitro* and *in vivo* determined with the inverse adding-doubling method. **(A)** Transmittance of each type of cornea; **(B)** absorption coefficient; **(C)** reduced scattering coefficient. HOC, human orthotypical cornea; HHC, human heterotypical cornea; HC, human artificial corneas (HOC and HHC). Controls are normal rabbit corneas (CTR). a: differences with controls were statistically significant; b: differences with HC *in vivo* at 3 m were statistically significant; c: differences with HOC *in vitro* were statistically significant; d: differences with HHC *in vitro* were statistically significant.

Tests of the optical behavior of controls and HOC and HHC bioengineered human corneas showed that light transmittance in all samples depended on the wavelength, and statistically significant differences were found among the different samples analyzed here. Globally, average values of bioengineered corneas represented approximately 60% of the transmittance in control corneas (approximately 80% for all HOC samples and 30% for al HHC samples) (Figure 7A). Interestingly, the duration of development in culture did not significantly influence the transmittance capacity of *in vitro* samples (*P* > 0.05). However, the type of sample was directly related with this parameter, and HOC *in vitro* samples showed significantly higher transmittance than HHC *in vitro* samples (*P* = 1.57 × 10^–272^). The absorption and reduced scattering coefficients values of HOC *in vitro* samples were significantly higher than controls (*P* = 2.31 × 10^–5^ for absorption and *P* = 1.81 × 10^–39^ for scattering), and the same behavior was found for HHC *in vitro* samples (*P* = 1.78 × 10^–122^ for absorption and *P* = 2.37 × 10^–199^ for scattering). However, HOC showed significantly lower levels of absorption and reduced scattering than HHC *in vitro* samples (*P* = 1.01 × 10^–204^ for absorption and *P* = 3.78 × 10^–285^ for scattering) (Figures 7B,C).

When HOC and HHC were grafted *in vivo*, we found a substantial increase in transmittance values. In HOC and HHC *in vivo* models 3 months after implantation, global mean transmittance was approximately 55 and 63%, respectively, corresponding to approximately 66 and 76%, respectively of the transmittance in control corneas. Therefore, we found an increase of transmittance 12 months after implantation, with transmittance values of approximately 75% for both HOC and HHC, corresponding to 90% of control corneas. Differences between HOC and HHC were marginally significant at this time (*P* = 0.00719), but both cornea models were significantly lower than controls (*P* = 1.82 × 10^–180^ for HOC and *P* = 2.96 × 10^–196^ for HHC). Regarding the absorption coefficient, we found that both HOC and HHC had significantly higher absorption coefficients than controls after 3 months of follow-up (*P* < 1.00 × 10^–100^ for HOC and HHC), with HOC being higher than HHC (*P* = 0.00001). However, *in vivo* samples 12 months after implantation showed absorption coefficients that were lower than those in control corneas, with differences being statistically significant (*P* < 1.00 × 10^–100^). Finally, we found that the reduced scattering coefficients of HOC and HHC after 3 months were lower than *in vitro* samples, although did not reach the low levels of control corneas (*P* < 1.00 × 10^–100^ for HOC and *P* = 4.84 × 10^–302^ for HHC). After 12 months, reduced scattering values were very similar to control corneas, although statistical differences with controls were detected (*P* = 1.58 × 10^–111^ for HOC and *P* = 2.36 × 10^–88^ for HHC). At this time, differences between HOC and HHC were marginally significant (*P* = 0.19009) for the reduced scattering.

## Discussion

The potential usefulness of novel HHC models lies in their ability to support the *in vitro* construction of human bioartificial cornea substitutes without the drawbacks associated with the use of autologous limbal stem cells (Behaegel et al., 2017). For this reason, we evaluated the *in vitro* and *in vivo* behavior of novel HHC substitutes generated from HWJSC.

Our results showed that both HHC and HOC cornea substitutes may hold potential for clinical use, and *in vivo* outcomes obtained with both cornea types showed no significant side effects on the host eyes. The initial inflammatory process found at 3 months was self-limited, and most cases evaluated at 12 months were devoid of significant inflammation. This was especially evident in HHC, which did not show any case of inflammation after 12 months. Although the differences with HOC were not substantial at this time (one case vs. no cases), we might hypothesize that the use of MSC in HHC could exert a positive influence in terms of inflammation. Even though future works should be carried out with a larger sample size to confirm or not this hypothesis, the immunomodulatory effects of HWJSC have been previously demonstrated in different settings (El Omar et al., 2014; Garzon et al., 2020). Analyses with OCT disclosed adequate biointegration of the grafted tissue with host cornea demonstrated by a close attachment of both tissue layers, albeit with a detectable limit between the two structures after 12 months. In this regard, it is well known that complete integration of tissues implanted in the cornea, including human keratoplasty, may progress slowly due to the avascular nature of the human cornea (Amiri et al., 2017). In fact, it has been demonstrated that a transplanted cornea could be easily detached by mild trauma or physical activity several years after the transplantation procedure (Jafarinasab et al., 2012).

In addition, our histological analyses confirmed previous reports (Garzon et al., 2014b) showing that HWJSC were able to differentiate *in vitro* into an epithelial-like layer on top of the stromal substitutes, although the histological and morphological patterns of both cornea types showed some differences that suggest that the differentiation process is not complete *in vitro* (Garzon et al., 2014b). However, the epithelial layer in both HOC and HHC corneas became similar to the native human cornea epithelium once were grafted *in vivo*. This finding suggests that the *in vivo* environment and the local release of soluble paracrine factors may be necessary to induce and enhance terminal cell differentiation of HWJSC (Alfonso-Rodriguez et al., 2015).

Analyses of the corneal stroma showed that the grafted bioengineered stroma substitute was integrated into the host cornea, with no evidence of rejection or other side effects at the histological level. Our results confirmed that stromal cells expressed vimentin, which is essential for normal growth, differentiation, integrity and function in corneal cells as previously demonstrated (Kivela and Uusitalo, 1998). In addition, our analyses confirmed the presence of mature collagen fibers in corneas grafted *in vivo*, and also in *in vitro* samples with the HHC model. The early expression of collagen by HHC cells may be explained by the high biosynthetic activity of WHJSC, which derive from a mucous connective tissue that is committed to produce large amounts of fibrillar and non-fibrillar extracellular matrix components in the umbilical cord (Corrao et al., 2013). In summary, the presence of vimentin-positive stromal cells immersed within a collagen-rich matrix resembling the native corneal stroma suggests that the implanted substitute may be undergoing adequate stromal differentiation after *in vivo* grafting. Moreover, the *in vivo* formation of a basement membrane on top of the stroma may support epithelial cell development, which appears to be a critical factor in corneal healing and transparency (Torricelli et al., 2016).

Analysis of the epithelial layer in the bioartificial cornea models demonstrated that HHC were able to develop a stratified epithelial-like structure similar to that seen in the HOC model. Strikingly, both HHC and HOC grafted *in vivo* showed high levels of expression of the cornea epithelium markers CK3/12 and PKG and several crystallins, similar to the levels seen in native human corneas. However, HHC showed late *in vitro* expression of both markers compared to HOC. These findings are not unexpected, since the HOC model was constructed with corneal cells that are previously committed to cornea differentiation, whereas HWJSC would likely require a differentiation induction process driven by different factors (Garzon et al., 2020). In addition to CK3/12 and PKG, further analyses should be carried out to determine the expression of other markers of corneal epithelial cells and limbal stem cells, such as KRT15, KRT17, ΔNp63, and ABCG2 (Ebrahimi et al., 2009; Wright and Connon, 2013). In this regard, we previously demonstrated that HOC show positive expression of most of these markers by microarray (Rico-Sanchez et al., 2019), (data are accessible at https://www.ncbi.nlm.nih.gov/geo/query/acc.cgi?acc=GSE86584), and that native HWJSC express high amounts of MSC undifferentiation markers, which decrease upon epithelial differentiation coinciding with a positive expression of specific differentiation markers (Alaminos et al., 2010). Whether or not this also occurs in our HHC model remains unexplored, although the positive results found with H&E, TEM, and SEM analyses support the hypothesis that HWJSC may have been able to differentiate to corneal epithelium.

An important unsolved question is the adequate characterization of the xenograft that allow us to definitely identify the origin of the cells found in the rabbit cornea. Rabbit limbal epithelial stem cells show rapid self-renewal (Zheng et al., 2019), and grafted human cells may disappear after several months of *in vivo* follow-up. In this regard, we found that two primary antibodies (anti-PKG and anti-CRY-λ1) showed positive expression in corneas grafted in rabbits, and very low expression in rabbit controls. Although this supports the possibility that the cells found in corneas grafted *in vivo* were human, survival of the grafted epithelial cells should be confirmed in future studies using FISH analysis or other highly sensitive techniques (Catanese et al., 2011).

Apart from the role of crystallins as corneal epithelium marker, a crucial aspect in the analysis of bioengineered human corneas is transparency, which is directly dependent on many factors such as the presence of cornea crystallins (Cvekl and Zhang, 2017). Although crystallins are not well known, it has been demonstrated that their contribution to cornea transparency is directly related to their molecular weight and three-dimensional structure, and their spatial structure can control light scattering (Tardieu, 1998). Transparency is essentially controlled by α crystallins, which are the biggest of these proteins and have adequate structure determining corneal transparency (Tardieu, 1998). Other crystallins with lower molecular weights such as crystallin β, λ, and Z could influence corneal transparency to a lesser extent. Analysis of crystallin expression should be therefore considered in corneas developed by tissue engineering. In the present study, we confirmed that bioengineered corneas maintained *in vitro* showed low expression of most cornea crystallins compared to human controls, as previously reported by our group (Garzon et al., 2014b). The low expression of CRY-αA and CRY-αB shown by *in vitro* samples coincided with the lowest levels of transparency (as determined by light transmittance) and the highest levels of light absorption and reduced scattering, especially in the first weeks of development, and suggest that the corneal epithelium differentiation level of these samples was low. Remarkably, the expression levels of collagen type I also tended to be low in corneas maintained *in vitro*, suggesting that an appropriate organization of the stromal fibers is crucial for corneal transparency (Meek and Knupp, 2015). In contrast, bioengineered HHC and HOC corneas grafted *in vivo* showed improved transparency after follow-up for 12 months. This would be expected to correlate with the higher expression of crystallin epithelial markers in our *in vivo* samples. Indeed, the levels of CRY-αA, CRY-αB, and CRY-Z became similar to control corneas in HHC, and the levels of CRY-αA and CRY-β were similar to control corneas in HOC. The transparency of *in vivo* corneas determined by light transmittance did not reach the values in native corneas, but was approximately 90% of controls after 12 months, with no differences between both models. Together, these findings suggest that bioengineered corneas require prolonged *in vivo* periods to synthesize and release key proteins related to transparency, such as collagen type I and several types of crystallins.

One of the limitations of the present study is the need of analyzing additional limbal stem cell markers in HOC and HHC by immunohistochemistry, along with the previously mentioned need of confirming the human nature of the epithelial layer. In addition, the bioengineered corneas generated in this work are devoid of the cornea endothelial layer, and future studies should be focused on the generation of full-thickness bioartificial corneas including the epithelial, stromal and endothelial corneal layers (Giasson et al., 2014).

## Conclusion

Altogether, our results suggest that bioartificial cornea models may show adequate *in vivo* biointegration levels, which may support their clinical use. HWJSC showed cornea epithelial differentiation potential and may be considered as a potential alternative epithelial cell source for the generation of human corneas by tissue engineering without the need of using epithelial cell cultures established from human cornea biopsies.

## Data Availability Statement

The datasets generated for this study are available on request to the corresponding author.

## Ethics Statement

The studies involving human participants were reviewed and approved by the Human Research and Ethics Committee of the province of Granada (PEIBA). The patients/participants provided their written informed consent to participate in this study. The animal study was reviewed and approved by the Ethics and Animal Experimentation Committee of the University of Granada and the Andalusian Directorate of Agricultural Production (CEEA).

## Author Contributions

IG, JC-A, MM-P, and MA carried out the artificial tissue development, data analysis, and manuscript preparation. CG-G and JC-A carried out the animal studies and obtained the clinical results. AI, JC, and MP were responsible for the optical analyses. MM was involved in immunofluorescence analyses in all samples. CC was responsible for TEM microphotographs. All authors contributed to the article and approved the submitted version.

## Conflict of Interest

The authors declare that the research was conducted in the absence of any commercial or financial relationships that could be construed as a potential conflict of interest.
